# Molecular delimitation of European leafy liverworts of the genus *Calypogeia* based on plastid super-barcodes

**DOI:** 10.1186/s12870-020-02435-y

**Published:** 2020-05-28

**Authors:** Monika Ślipiko, Kamil Myszczyński, Katarzyna Buczkowska, Alina Bączkiewicz, Monika Szczecińska, Jakub Sawicki

**Affiliations:** 1 Department of Botany and Nature Protection, Faculty of Biology and Biotechnology, University of Warmia and Mazury in Olsztyn, Olsztyn, Poland; 2 Department of Biology, Institute of Experimental Biology, Adam Mickiewicz University in Poznań, Poznań, Poland

**Keywords:** Super-barcoding, DNA barcode, *Calypogeia*, *ndhB*, *ndhH*, *trnT-trnL*

## Abstract

**Background:**

Molecular research revealed that some of the European *Calypogeia* species described on the basis of morphological criteria are genetically heterogeneous and, in fact, are species complexes. DNA barcoding is already commonly used for correct identification of difficult to determine species, to disclose cryptic species, or detecting new taxa. Among liverworts, some DNA fragments, recommend as universal plant DNA barcodes, cause problems in amplification. Super-barcoding based on genomic data, makes new opportunities in a species identification.

**Results:**

On the basis of 22 individuals, representing 10 *Calypogeia* species, plastid genome was tested as a super-barcode. It is not effective in 100%, nonetheless its success of species discrimination (95.45%) is still conspicuous. It is not excluded that the above outcome may have been upset by cryptic speciation in *C. suecica*, as our results indicate. Having the sequences of entire plastomes of European *Calypogeia* species, we also discovered that the *ndhB* and *ndhH* genes and the *trnT-trnL* spacer identify species in 100%.

**Conclusions:**

This study shows that even if a super-barcoding is not effective in 100%, this method does not close the door to a traditional single- or multi-locus barcoding. Moreover, it avoids many complication resulting from the need to amplify selected DNA fragments. It seems that a good solution for species discrimination is a development of so-called “specific barcodes” for a given taxonomic group, based on plastome data.

## Background

The genus *Calypogeia* Raddi is one of the four genera of the family Calypogeiaceae H. Arnell belonging to liverworts (Marchantiophyta). Liverworts are one of three divisions (besides mosses and hornworts) of plants known as bryophytes, and comprise about 7, 000 species in the world [1]. Liverworts are organisms that played a key role in land plants evolution. As fossil evidence suggests liverworts were among the first land plants and present on land approximately 475 million years ago [2–4]. The hypothesis, that liverworts are one of the earliest-diverging group of land plants is supported by phylogenetic analyses based on large molecular data sets [5–8]. The family Calypogeiaceae forms one of the youngest clade of leafy liverworts (subclass Jungermannidae) with divergence time estimated at ca. 50 million years [9]. The genus *Calypogeia* is the only genus of this family occurring in Europe. It comprise over 90 described species [10, 11]. However, the highest species diversity of the genus was recorded in the tropics [12]. In the Holarctis, species richness of *Calypogeia* is much lower and represented by only 9–13 species in its different parts. In Europe, there are only nine species of *Calypogeia*: *C. azurea* Stotler & Crotz, *C. integristipula* Steph., *C. neesiana* (Massal.& Carestia) M üll. Frib., *C. suecica* (Arnell & J.Perss.) M üll. Frib., *C. muelleriana* (Schiffn.) M üll. Frib., *C. sphagnicola* (Arnell & J.Perss.) Warnst. & Loeske, *C. fissa* (L.) Raddi, *C. arguta* Nees & Mont. and *C. azorica* Bischl., which is endemic for Islands of Macaronesia [13, 14]. Some of the species e.g. *C. azurea*, *C. suecica*, *C. sphagnicola*, *C. integristipula*, *C. neesiana* have wider distribution in northern hemisphere, and are reported from North America, Europe and Asia [15–17].

In liverworts, the dominant phase of the life cycle is the haploid gametophyte. The simple gametophyte morphology provides a limited number of diagnostic features therefore liverworts are, taxonomically, a difficult group. For these reasons, identification of species, genera/subgenera classification as well as phylogenetic analysis based only on morphological characteristics is difficult and often ambiguous [18].

Rapidly developing molecular biology and bioinformatics gives the possibility of widespread use of DNA sequences in taxonomic studies. DNA barcoding proposed by Hebert et al. [19], is already commonly used for correct identification of difficult to determine species, to reveal cryptic species, or detecting new taxa [20–22]. Unfortunately, it was not possible to select universal single- locus barcode for plants, that would be sufficient to distinguish in closely related taxa. Thus, two-locus combination (*rbcL* + *matK*) was recommended as the best plant barcode by CBOL Plant Working Group [23]. However, not in all plant groups, especially in liverworts, this combination works well with universal primers due to difficulties with amplification of the *matK* locus [24, 25]. High-throughput sequencing technology offers new opportunities for using genomic data in the study of biological diversity of various plant species – super- barcoding [26, 27]. Thus, the plastid super- barcode is a whole- plastid genome sequence using in plant identification, particularly in situations, when single- or multi- locus barcodes fail. The whole plastid genomes and nuclear rRNA cluster were proved to be highly useful for distinguishing closely related species [28–30], varieties and individual genotypes [31] or cryptic species [32]. Recently, the whole plastid and mitochondrial genomes have been increasingly used in the phylogenetic analysis at different taxonomic levels [33–37]. The analysis of complete genome sequences is the way to detect parts of the genome, which have optimal variation for individual group of plants and can act as specific barcodes [38]. Although one of the first chloroplast genome was sequenced from liverwort *Marchantia paleacea* Bertol. almost 30 years ago [39], the number of liverwort species with complete genomes, compared to vascular plants, currently available in databases is still very scarce. Until now, whole plastid genomes have been sequenced for 21 liverwort genera and for 23 species [40].

Only a few species of the *Calypogeia* genus were analyzed at molecular level to date. Isozyme and molecular markers revealed that some of the European *Calypogeia* species described on the basis of morphological criteria are genetically heterogeneous and, in fact, are species complexes that consist of previously unrecognized species, e.g. *C. fissa* [41], *C. muelleriana* [42, 43], *C. sphagnicola* [44]. Studies based on isozyme polymorphism, cytology and flow cytometry indicate that hybridization and genome duplication are important processes of speciation in the *Calypogeia* genus [45, 46]. The comparative analysis of complete mitogenomes of four *Calypogeia* species revealed unexpected losses of introns [47], showing that molecular resources of liverworts are still unexplored. The molecular studies of the genus concerned the selected species and were based on the small fragments of chloroplast genome [44].

In the present study we: *i*) established and characterized the organization of the complete chloroplast genome sequence of European *Calypogeia* species; *ii*) identified and evaluated the various DNA barcodes for *Calypogeia* species *iii*) analysed the phylogenetic relationships between European species of the genus *Calypogeia*.

## Results

### Structure and sequence variation of the Calypogeia plastid genome

**Fig. 1:**
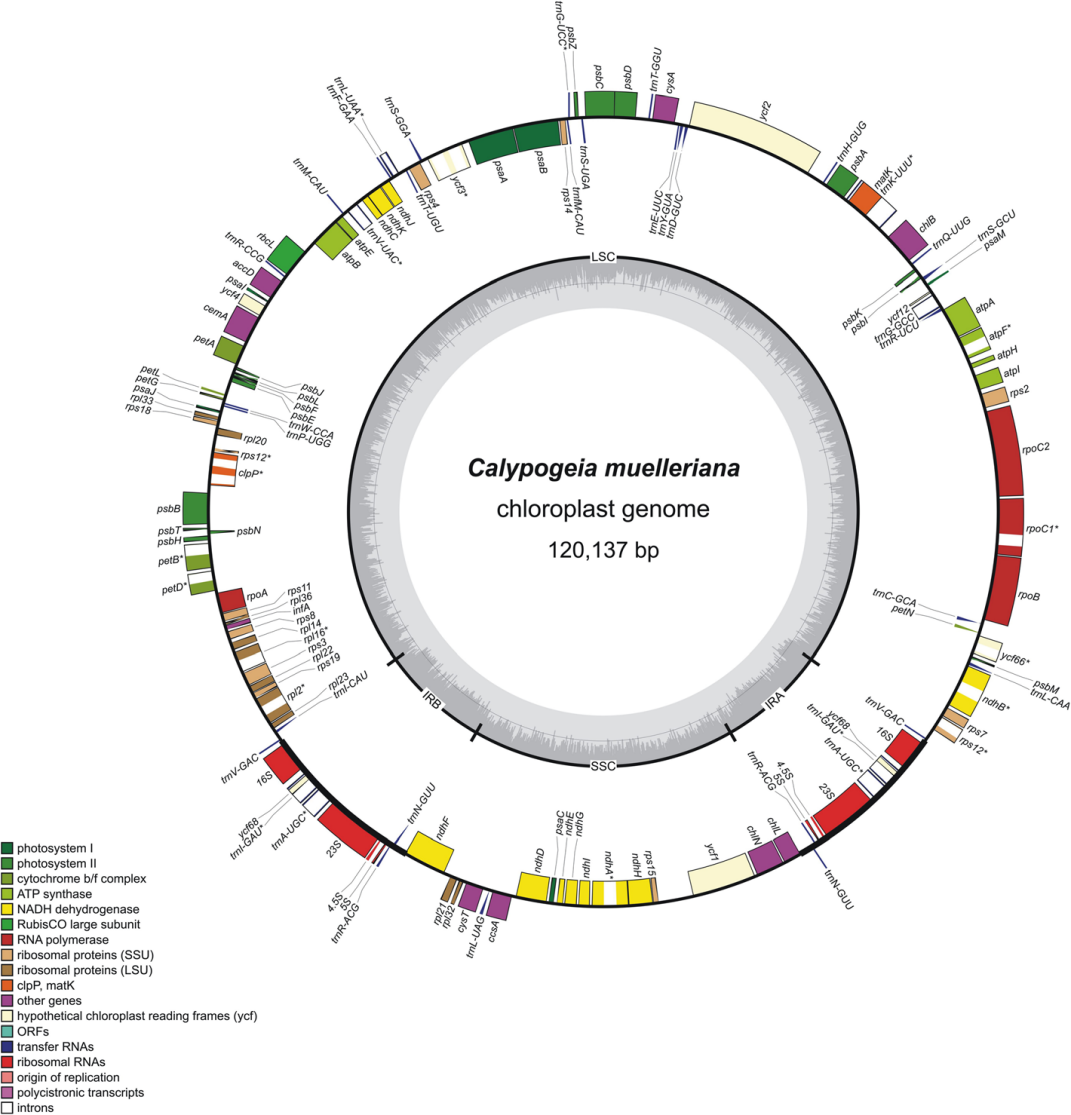
Gene map of the *Calypogeia muelleriana* chloroplast genome. Genes inside and outside the outer circle are transcribed in counterclockwise and clockwise directions, respectively. The genes are color-coded based on their function. The dashed area in the inner circle visualizes the G/C content. Pseudogenes have been marked with Ѱ

Plastid genome of *Calypogeia* is a circular molecule consisted of typical regions for land plants: a large single copy (LSC) ranging from 82, 377 bp in *C. arguta* to 83, 289 bp in *C. muelleriana*, a small single copy (SSC) ranging from 19, 933 bp to 20, 016 bp in *C. arguta* and two inverted repeat regions (IRs) in the range from 8, 236 bp in *C. muelleriana* to 8, 674 bp in *C. azorica* (Fig.1). One hundred twenty-two unique genes (taking into account only one copy of inverted repeat regions) were identified in the plastome of *Calypogeia*: 81 protein-coding genes, four ribosomal RNAs, 31 transfer RNAs and six *ycf* genes of an indeterminate function. One gene of *ycf* family (*ycf68*) in IR region was annotated as a pseudogene (marked with Ѱ in Fig.1). In the whole chloroplast genome of *Calypogeia* 20 introns have been identified. Ten protein-coding genes: *rps12*, *ndhA*, *ndhB*, *rpl2*, *rpl16*, *rpoC1*, *ycf66*, *atpF*, *petB*, *petD* and six transfer RNAs: *trnI-GAU*, *trnA-UGC*, *trnV-UAC*, *trnL-UAA*, *trnK-UUU*, *trnG-UCC* contained a single intron, while *clpP* and *ycf3* harbored two introns. The base composition of cpDNA (chloroplast DNA) was the following: A (32.3%), C (17.6%), G (17.7%), T (32.3%) with an overall GC content at the level of 34.6%.

**Fig. 2:**
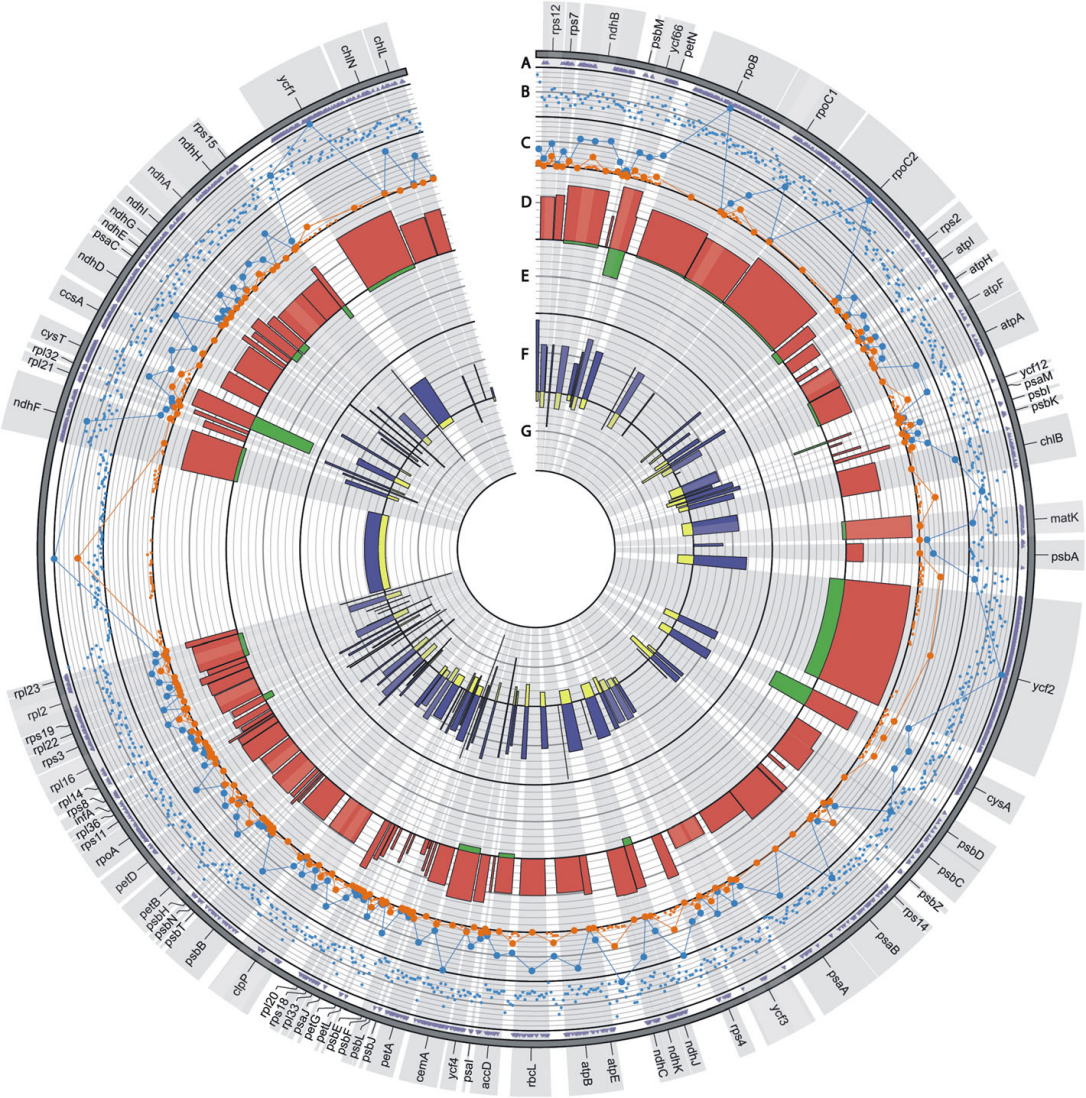
SNP and indel variation among plastomes of *Calypogeia*. Track A shows nonsynonymous SNP occurrence within genes. Track B and C represent identified SNPs (small blue dots) and indels (small orange dots) per 100 bp window size (maximum value = 40). Line plot, comprising B and C track, represents SNPs (blue line) and indels (orange line) within each exon, intron or intergenic spacer (snp max. Value = 400, indel max. Value = 100). Track D represents percent of SNPs per CDS length (maximum value = 22) while track E represents percent of indels per CDS length (maximum value = 2). Track F represents percent of SNPs per noncoding region length (max. Value = 30) while track G represents percent of indels per noncoding region length (max value = 20)

The chloroplast genome length of *Calypogeia* genus ranged from 119, 628 bp in *C. arguta* to 120, 170 bp in *C. muelleriana* (Table S1). The alignment of 22 specimens representing 10 *Calypogeia* species was 122, 472 bp in length and its pair identity was 95.6%. Sequence variability was caused by the presence of SNP and indels, but the number of SNPs was predominant. In coding and non-coding regions of plastome 737 indels and 15, 666 SNPs were identified, of which over 36% were nonsynonymous (Fig. 2., S2, S3). The percent of polymorphic sites (P_%_) for plastome of *Calypogeia* was 13.67%, while a mean value of π was 0.035076.

Analysis of genetic variability within plastome of *Calypogeia* revealed that the most variable protein-coding region with length over 100 bp was *cysT* gene (Table 1, S2), not related to photosynthesis. It was characterized with 20.65% of polymorphic sites (P_%_) and π = 0.07389. This 867 bp long region contained 15 indels and as many as 164 SNPs, where 92 of them were nonsynonymous. The second, fourth and fifth most variable protein-coding regions were the genes belonging to the *ycf* family with an undefined function: *ycf2*, *ycf66* and *ycf1* respectively. The percent of polymorphic sites for these genes ranged from 17.54 to 20.17 (π = 0.04912–0.07282). The third high position in the ranking of the most variable protein-coding regions took the *matK* gene with 218 SNPs and one indel (P_%_ = 19.89, π = 0.05696) (Table 1, Table S2). The mentioned genes: *cysT*, *matK*, *ycf1*, *ycf2* genes correctly distinguished as many as 21 out 22 sequences by assigning them to the appropriate species, whereas discriminatory power of the *ycf66* gene was slightly lower and was 90.9% (Table 1.). The percent of polymorphic sites (P_%_) for protein-coding sequences ranged from 2.5 to 20.65, while π value stretched from 0.00474 to 0.07389 (Table S2).

**Table 1:**
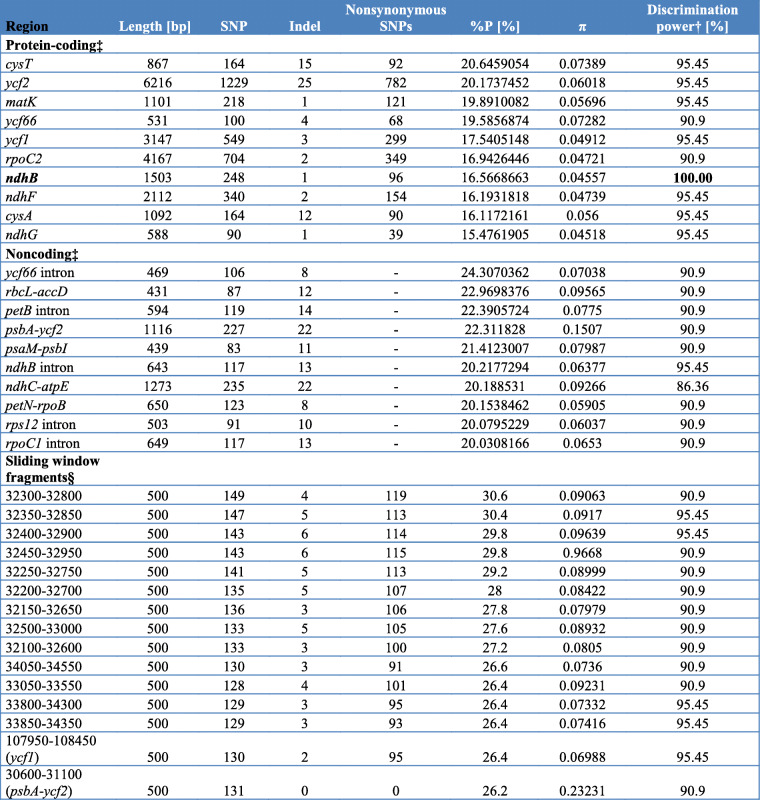
The top of the most variable chloroplast regions of *Calypogeia* species.

Among non-coding regions longer than 100 bp the highest variability was present in *rpl14*-*rpl16* spacer (121 bp, P_%_ = 29.75, π = 0.12371). In this region 30 SNPs and 6 indels were identified. The second most polymorphic region, between *rpl32* and *cysT* genes, had 28.93% of polymorphic sites and π = 0.09156. The slightly less variable non-coding region was *atpH*-*atpF* spacer with the P_%_ value at the level 28.89% and π = 0.11377 (Table S3). However, all of them as well as fragments being among the top ten most non-coding regions did not distinguish the tested species.

The most polymorphic 500 bp-long nucleotide fragments of plastome, determined artificially without considering their biological function, had a variability similar to non-coding regions. The percent of polymorphic sites for 13 most mutable fragments stretched between 26.4–30.6%, whereas π value for the same fragments ranged from 0.07332 to 0.09668 (Table S4). All these fragments belonged to the *ycf2* gene and properly discriminated 20–21 out of 22 sequences, classifying them to the appropriate *Calypogeia* species (Table 1). On the other hand, 10 most polymorphic 500 bp fragments, taking into account only the nucleotide diversity (π = 0.1533–0.23231), were present within *psbA*-*ycf2* spacer that correctly assigned 20 out of 22 sequences to the appropriate *Calypogeia* species.

### Phylogeny

**Fig. 3:**
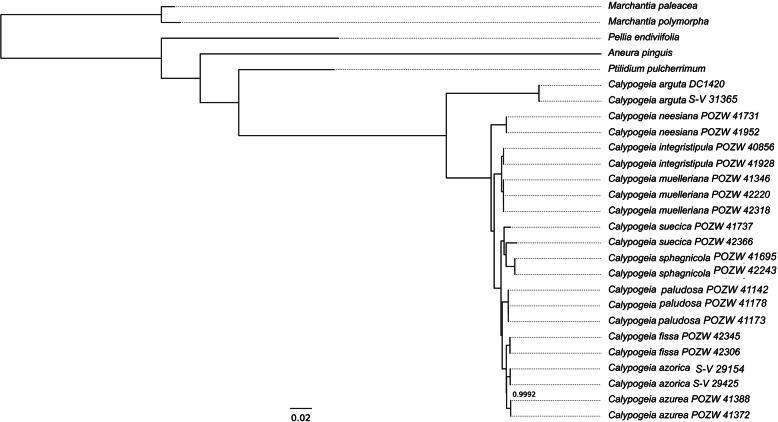
CDS-based phylogram derived from a Bayesian analysis. The posterior probability value lower than 1 is given at the node

The alignment of the set of chloroplast CDS was used to construct the phylogenetic tree (Fig. 3). Trees based on the whole plastomes and amino acid sequences were very similar to each other and to the CDS-based phylogram (Fig. S1). The analysis inferred from the partitioned chloroplast CDS dataset clearly distinguished almost all species of liverworts with strong branch support. All duplicate *Calypogeia* species are correctly paired on the phylogenetic tree. The most separated species from other *Calypogeia* species on the phylogenetic tree is *C. arguta*, as expected. Still the most interesting is the position of *C. suecica* which is grouped with *C. sphagnicola*. Even more importantly the distance between *the C. suecica* species pair is by far the greatest of all *Calypogeia* pairs which may suggest that the two *C. suecica* species are in fact two distinct species.

### Species delimitation

The Poisson Tree Processes (PTP) analysis grouped 22 *Calypogeia* individuals into 11 species, what exceed the number of species used in this study by one. The PTP analysis classified two specimens of *C. suecica* as two different species. The support for species division was pretty high and was ranged from 0.922 (for *C. arguta*) to 1.00 (for *C. suecica*) (Table 2). The above results are reflected in phylogenetic trees (Fig. 3, Fig. S1), where representatives of each species are grouped together belonging to the same clade, while individuals of *C. suecica* are separated from each other and form a shared clade with *C. sphagnicola*. Pairwise identity for two plastomes of *C. suecica* reached the value of 97.7%, whereas in other species was between 99.6% (for *C. neesiana*) and 99.99% (for *C. fissa*).

**Table 2:** Species delimited from set of analyzed individuals by PTP simple heuristic search

Species delimited by PTP analysis	Posterior delimitation probability
*C. suecica POZW 41737*	1.0
*C. suecica POZW 42366*	1.0
*C. azurea POZW 41372,* *C. azurea POZW 41388*	0.999
*C. sphagnicola f. sphagnicola POZW 41695,* *C. sphagnicola f. sphagnicola POZW 42243*	0.999
*C. neesiana POZW 40856,* *C. neesiana POZW 41952*	0.999
*C. fissa POZW 42306,* *C .fissa POZW 42345*	0.998
*C. sphagnicola f. paludosa POZW 41142,* *C. sphagnicola f. paludosa POZW 41173,* *C. sphagnicola f. paludosa POZW 41178*	0.998
*C. integristipula POZW 40856,* *C. integristipula POZW 41928*	0.992
*C. azorica S-V 29154,* *C. azorica S-V 29425*	0.991
*C. arguta DC1420,* *C. arguta S-V 31365*	0.922
*C. muelleriana POZW 41346,* *C. muelleriana POZW 42220,* *C. muelleriana POZW 42318*	0.891
*C. muelleriana POZW 41346,*	0.125
*C. muelleriana POZW 42220*	0.125
*C. muelleriana POZW 42318*	0.125
*C. arguta DC1420*	0.078
*C. arguta S-V 31365*	0.078
*C. azorica S-V 29154*	0.009
*C. azorica S-V 29425*	0.009
*C. sphagnicola f. paludosa POZW 41142*	0.002
*C. sphagnicola f. paludosa POZW 41173*	0.002
*C. sphagnicola f. paludosa POZW 41178*	0.002
*C. fissa POZW 42306*	0.002
*C .fissa POZW 42345*	0.002
*C. neesiana POZW 40856*	0.001
*C. neesiana POZW 41952*	0.001
*C. sphagnicola f. sphagnicola POZW 41695*	0.001
*C. sphagnicola f. sphagnicola POZW 42243*	0.001
*C. azurea POZW 41372*	0.001
*C. azurea POZW 41388*	0.001

**Fig. 4:**
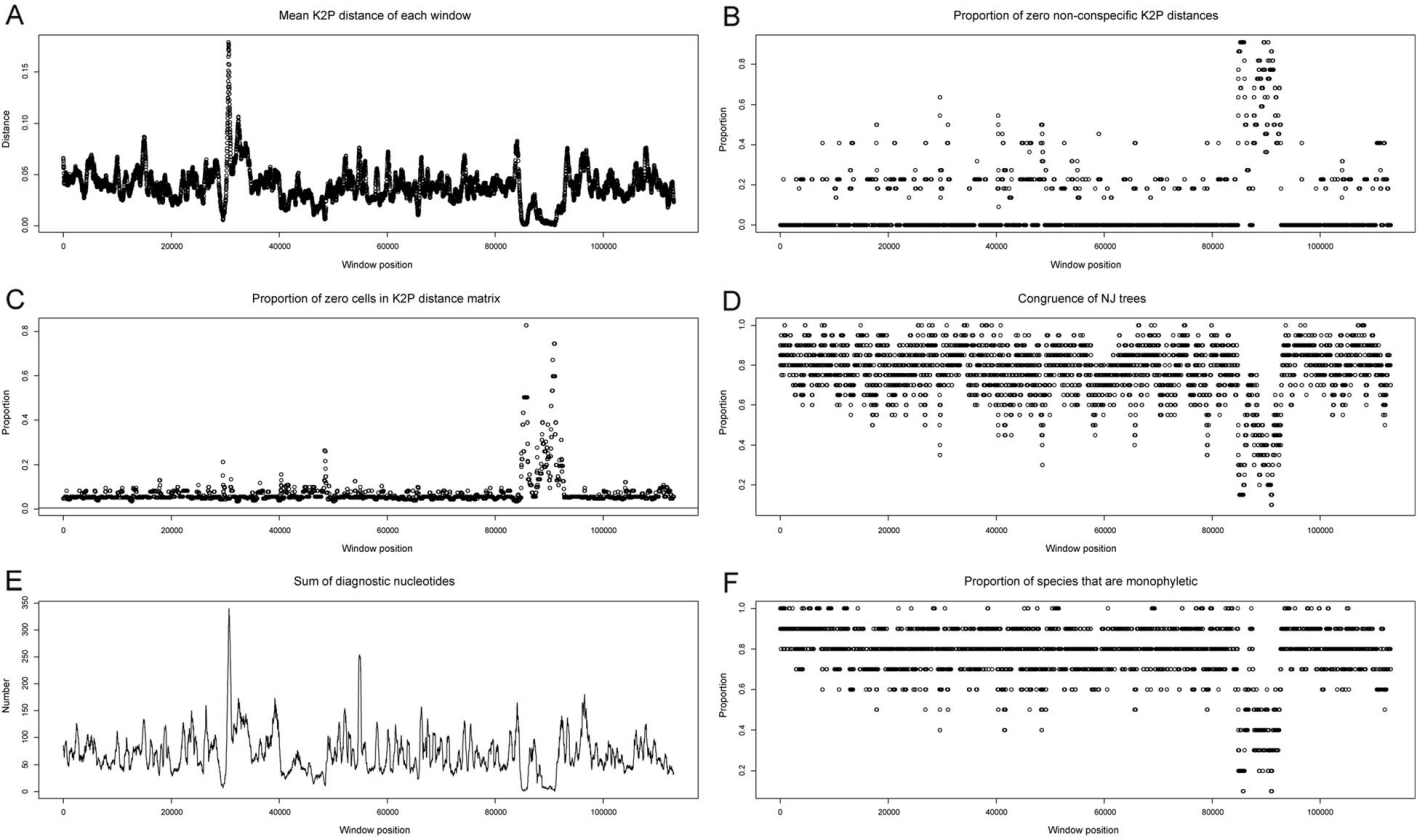
Results of several analyzes across the plastome sequences of *Calypogeia* sp. using the sliding window method. **a**- the plot of the mean Kimura 2-parameter distance matrix for each 500 bp- long window. The greatest one is at about 30, 000 bp position and the lowest one at the 90, 000 bp position. **b**- the proportion of zero non- conspecific distances, which find their maximum at around 90, 000 bp. **c**- the proportion of zero cells in the distance matrix. This is maximized around 90, 000 bp. The unbroken horizontal line crossing the y-axis at 0 is the proportion of zero cells in the distance matrix created from the full dataset. **d**- the proportion of clades that are identical between the windows and the full dataset. It is pretty high at most windows and visibly low at around 90, 000 bp position. **e**- the sum of diagnostic nucleotide positions for all species. The most of these nucleotides are at around 30, 000 bp position and the least at around 90, 000 bp position. **f**- the proportion of species that are monophyletic. The position around 90, 000 bp is clearly in the doldrums, but most positions distinguish species pretty well.

**Fig. 5:**
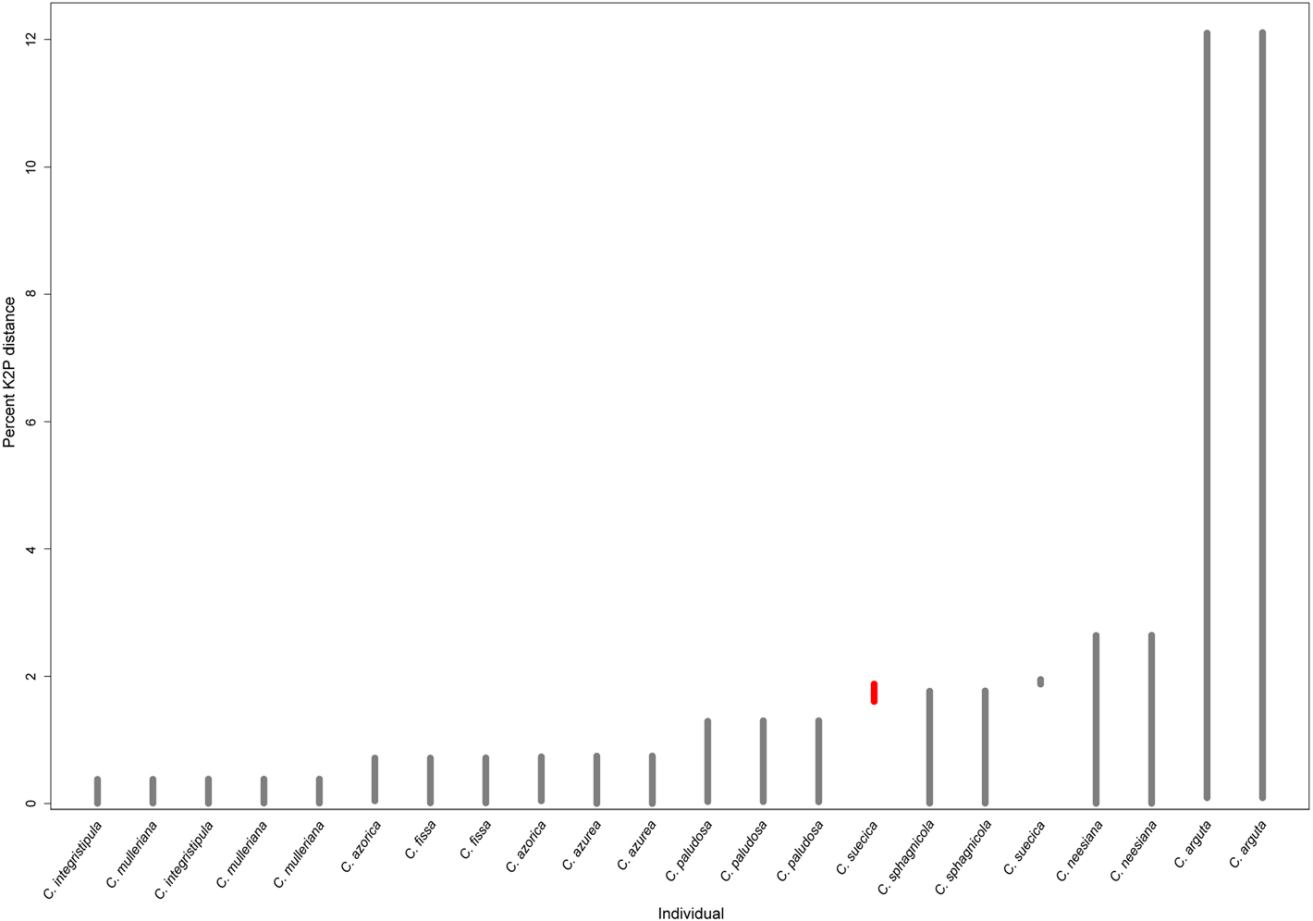
Lineplot of the barcode gap for the 22 *Calypogeia* species. For each individual in the dataset, the grey lines represent the furthest intraspecific distance (bottom of line value), and the closest interspecific distance (top of line value). The red lines show where this relationship is reversed (no barcoding gap)

The chloroplast genomes of all species differed from each other. The strongest differences occurred between *C. arguta* and other *Calypogeia* species (Fig. 3, Fig. S1), where the average interspecific distance was 0.121079 and the number of fixed nucleotide differences ranged from 11, 329 (between *C. arguta* and *C. suecica*) to 12, 414 (between *C. arguta* and *C. sphagnicola*). The largest number of indels distinguishing species was 687 and occurred between *C. arguta* and *C. azorica*. The most similar species turned out to be *C. muelleriana* and *C. integristipula* differing only in 412 SNPs and 428 indels. However, these differences were sufficient to identify the aforementioned species as showed both in Spider and Species Identifier analyzes (Fig. 4a-f, Table 1.). According to these programs whole chloroplast genomes of *Calypogeia* species can be used as a super- barcode with a success of 95.45%. Spider analysis demonstrated that only a plastome of *C. suecica* cannot be employed as a super- barcode. For one specimen of this species there existed no barcoding gap (Fig. 5.). Similarly, Species Identifier revealed that one specimen of *C. suecica* was incorrectly identified and mistaken for *C. paludosa*. However, the analysis of the most variable DNA coding fragments showed that there was one gene which sequence allowed for proper species identification of all analyzed plastome sequences, namely the *ndhB* gene (Table 1.). Among 10 most variable protein-coding sequences, seven genes allowed to correctly match to the species over 95% of analyzed sequences (Table 1.). The non-coding plastome sequences of *Calypogeia* were less effective. Only *ndhB* intron properly identified 21 out of 22 analyzed sequences and eight other non-coding fragments correctly assigned to the species over 90% of studied sequences (Table 1).

The analysis of sliding window also revealed high discrimination power of the studied species set, albeit lower than in the case of protein-coding sequences (Table 1). Out of 10 the most variable 500 bp-long fragments two correctly identified 21 sequences, while among the 10 most variable protein coding regions as many as eight allowed to properly distinguish 21 out of 22 sequences. All the 10 most variable 500 bp-long chloroplast DNA pieces belonged to the fragment of the *ycf2* gene. Within the 18 most polymorphic fragments generated by sliding window analysis, only two represented other regions of plastid DNA: a fragment of the *ycf1* gene and a fragment of the *psbA*-*ycf2* spacer. These results are also graphically presented in the Spider charts (Fig.4a-f), which indicate that the best chloroplast region for creating barcodes is around 30, 000 bp position, occupied by the *ycf2* gene.

Testing the usefulness of plant barcodes appearing in literature, we confirmed high efficiency of these DNA fragments in discrimination of *Calypogeia* species (discrimination power between 81.81–100%; Table S5). The sequences of *ndhH* gene and *trnT-trnL* spacer properly identified analyzed species in 100%.

## Discussion

### Comparative plastid genomics

Sequencing of plastid genomes of liverworts is gaining ground. Today, in the GenBank database the complete plastome sequences of five complex thalloid liverworts can be found, three of simple thalloid liverworts and 14 plastomes of leafy liverworts. Here we present 22 newly sequenced chloroplast genomes of 10 *Calypogeia* species (leafy liverworts). The structure of the above plastomes was typical for most plants and was divided into two IRs regions separated by LSC and SSC regions (Fig. 1.). The length of chloroplast genomes seems to be variable not only at the genus level, but also at the species and even at the individual level. Plastome of *Calypogeia* is 824–1, 366 bp shorter than the longest known leafy liverwort chloroplast genome of *Gymnomitrion concinnatum* [48] and 1, 880–2, 422 bp shorter than the longest known thalloid liverwort chloroplast genome of *Dumortiera hirsuta* [49]. *Calypogeia* species also varied in plastid genome length similarily to *Aneura pinguis* cryptic species [32]. Moreover, it was observed that plastome lengths differ within one species (Table S1). Similar event was recorded in the case of *Marchantia polymorpha subsp. ruderalis*, for which two independent research team obtained two different chloroplast genome lengths: 120, 457 bp [50] and 120, 304 bp [51].

The GC content of *Calypogeia* plastome is 34.6% and is almost the same like in *Gymnomitrion concinnatum* (34.5%), the leafy liverwort species for which the complete chloroplast genome was sequenced most recently [48]. The GC content in *Calypogeia* plastid genome falls in the range of values known for other liverwort species, from 28.8% for *Marchantia paleacea* [39] to 40.6% for *Aneura mirabilis* [52].

The comparative analysis of three known leafy liverwort plastomes (*Ptilidium pulcherrimum*, *Gymnomitrion concinnatum* and sequenced here *Calypogeia* sp.), revealed similarity in the gene content and order. All of them contain 122 unique genes: 81 protein-coding, 6 of unknown function (*ycf*), 31 tRNAs and 4 rRNAs. However, in *Ptilidium pulcherrimum cysA* (in LSC region) and *cysT* (in SSC region) are pseudogenes [53], whereas in the chloroplast genomes of *Calypogeia* and *Gymnomitrion concinnatum* as well as in complex thalloid liverworts (*Marchantia paleacea*, *Marchantia polymorpha*, *Dumortiera hirsuta*), these genes are functional. The *ycf68* motif that has been annotated as a pseudogene in *Calypogeia* plastid genome. In other liverwort species is also registered as a nonfunctional gene (e.g. *Aneura mirabilis*, *Gymnomitrion concinnatum*) or skipped in the plastome description (e.g. *Pellia endiviifolia*, *Aneura pinguis*, *Dumortiera hirsuta*, *Marchantia sp.*). On the other hand, Forrest et al. [53] are confused about the functionality of the hypothetical *ycf68* gene in *Ptilidium pulcherrimum*. In many vascular plants the above mentioned motif is reported, however as functional gene only in several lineages: *Stipa sp.* [28], *Lolium multiflorum*, *Festuca pratensis* [54]. Raubeson et al. [55] suggested that *ycf68* could be a pseudogene, but the occurrence of RNA editing in chloroplast genomes of many plants may result in restoring of fully worked gene.

### Phylogenetic relationships

The phylogenetic relationships between studied *Calypogeia* species obtained on the basis of the whole chloroplast genomes analysis are, in general, consistent with previous studies of the genus *Calypogeia* [56]. The whole plastome genomes analysis confirmed a close relationship of *C. muelleriana* and *C. integristipula* (Fig. 3.). Previous studies [41, 46] revealed that *C. muelleriana* is an allopolyploid, while *C. integristipula* is a haploid species, thus it can be assumed that *C. integristipula* was one of *C. muelleriana* parents who was the donor of its chloroplast genome. *C. sphagnicola* and *C. paludosa*, which originally were considered as the forms of *C. sphagnicola* i.e. *C. sphagnicola* f. *sphagnicola* and *C. sphagnicola* f. *paludosa* [57, 58] belong to two different clades, which support the hypothesis that they represent genetically distinct species [44]. *Calypogeia sphagnicola* belongs to the same clade as *C. suecica* (both haploid species), while allopoliploid species *C. paludosa* forms its own distinct clade, which is a sister to clade containing *C. azurea*, *C. azorica* and *C. fissa*. Moreover, our studies revealed a high variation of *C. suecica*, indicating the cryptic speciation within this species. *C. suecica* is an obligate xylicole, it is almost restricted to moist decorticated logs and shows low morphological variability [57]. However, in Europe two cytoforms of *C. suecica n* = 9 and *n* = 18 are reported by Lorbeer [59] and Paton [60] respectively, which may support the hypothesis that an unrecognized species is present within *C. suecica*. Our results indicate that *C. suecica* requires further molecular and morphological studies.

### Hot-spots and DNA barcoding

Analysis of the variability of the whole liverwort chloroplast or mitochondrial genomes rarely appears in studies. Hitherto, this type of analysis was carried out only among cryptic species of *Aneura pinguis* [32]. Therefore our results, obtained for a group of species belonging to one genus, are difficult to compare with the outcomes for the complex species of *A. pinguis*.

The research results for *A. pinguis* have shown that among protein coding regions genes *ycf1* and *ycf2* are ones of the most variable genes [32]. Similarly, in our studies on *Calypogeia*, the *ycf* genes: *ycf1*, *ycf2*, *ycf66* were in the top five of the most variable coding regions, what predisposes them to be a potential DNA barcodes. In the past few years, it is more and more often reported about the usefulness of the *ycf1* and *ycf2* genes in the identification of plant species [29, 61–63]. Especially two regions of the *ycf1* gene: *ycf1a* and *ycf1b* are highly variable and can serve as an effective barcode for land plants. *Ycf1*b fragment is proven to work better than any of the *matK*, *rbcL* and *trnH-psbA* applied individually and slightly better than the combination of *matK* and *rbcL* in woody plants [64]. On the other hand, the application of the *ycf1a* fragment in the discrimination of *Paris* species was more effective than the using only the *ycf1b* gene piece [65]. Nevertheless, a discrimination success of the *ycf1b* fragment (about 72%) in research by Dong and others [64] and the *ycf1a* gene piece both separately (52.63%) and in a combination with the *ycf1b* (89.47%) [65] was smaller than an application of the entire *ycf1* gene sequence in our studies for *Calypogeia* species (over 95%). However, wanting to use *ycf1* and *ycf2* genes as barcodes, one should keep in mind the limitations of these sequences in an amplification. The above mentioned genes are quite long (e.g. *ycf1*–3, 147 bp and *ycf2*–6, 216 bp in *Calypogeia*) and recovering the entire sequences of these genes in a PCR reaction would be a challenge. Not without reason Dong et al. [64] applied as barcodes for woody plants only the most variable and with the biggest resolution power the *ycf1* fragments. Our results indicated the most promising *ycf2* 500 bp-long fragments for *Calypogeia* species delimitation. As many as the first 13 positions in the list of the most-variable fragments of *Calypogeia* plastome with a length of 500 bp were taken by fragments of the *ycf2* gene (Table 1) and could be potential DNA barcodes. The discriminatory power of the 10 most-variable protein- coding regions in genus *Calypogeia* was in the most cases at the high level of 95.45% (Table 1). Only the resolution power for *ndhB*, *ycf66* and *rpoC2* genes had different values: 100, 90.9 and 90.9%, respectively. While the *ndhB* gene rather occurs commonly in plant chloroplast genomes, the *ycf66* gene not necessarily. A presence of this *ycf* gene was not reported in *Aneura mirabilis* [52] and *Aneura pinguis* [32]. Similarly, an occurrence of the *cysT* gene, the most variable protein- coding sequence in *Calypogeia*, is changeable in liverworts. The aforementioned gene is lacking, for example, in *Ptilidium pulcherrimum* [53] and *Aneura* species [32, 52]. *CysT* gene in above mentioned species functions as a pseudogene, therefore high variability is here justifiable [28]. Nevertheless, a literature does not mention the *cysT* and *ycf66* genes as DNA barcodes. Also, no one has reported the *ndhB* gene as an effective plant barcode, but it seems to be one of the core sequence in a species discrimination in *Calypogeia*. On the other hand, Krawczyk et al. [28] pointed out the potential of the *ndh* gene family in species identification, indicating the *ndhH* gene as the best performing loci for *Stipa*. Although *ndhH* was not listed at the top of the most variable coding regions in *Calypogeia* chloroplast genome (and therefore not tested in our research), its discrimination power was 100%. Slightly less, however also quite effective in the species identification was the *rpoC2* gene- reported to belong to the relatively fast evolving *rpo* genes [66]. The last statement was confirmed in our analyzes by the high polymorphism of this sequence (Table 1). Recently, reports on the *rpoC2* gene as a barcode are more frequent [22, 67–69].

Consortium for the Barcode of Life (CBOL) Plant Working Group recommended two locus: *rbcL* and *matK* as core DNA barcodes for plants [23]. In our research the *matK* gene was the third on the list of coding regions with the highest variability and correctly identified 21 out of 22 sequences. Unfortunately, the *matK* gene is said to be troublesome in amplification among bryophytes and ferns [23, 70, 71]. Therefore, it is inconclusive whether the use of the *matK* gene in the identification of species can be extended to bryophytes [71]. In contrast to the above, the PCR success of the *rbcL* gene is reported to be high [23, 70, 71]. However, it is mediocre in its capacity to distinguish specimens at the species level [23]. Despite the fact that the *rbcL* gene was not among the most variable coding regions in *Calypogeia*, the discriminatory power of the *rbcL* gene (90.9%) was almost the same as that of *matK* gene (Table 1, Table S2). The high resolution power of the *rbcL* was also reported among species of bryophytes [21, 70, 71] as well as its potential as a barcoding marker for bryophytes was noticed by some researchers [25, 32, 72]. However, Stech and Quandt [73] state that in general for bryophytes the *rbcL* gene exhibits low variation at the family level and therefore it is not useful for DNA barcoding among the early land plants. In our tests, the application of a two-locus barcode *rbcL* + *matK* did not raise the discriminatory power which was the same as for the *matK* individually (Table 1).

Liu et al. [70] also mentioned *rpoC1* and *rps4* regions as good potential barcodes for mosses. Actually, the resolution success for these sequences in the case of *Calypogeia* was considerable (95.45%; Table S5).

Among non- coding regions the resolution success of 100% in genus *Calypogeia* gave the *trnT-trnL* spacer, previously tested in the tribe Stipeae [28, 74, 75]. However there, this spacer as a separate region was not variable enough to give satisfying results. In the literature the *trnT-trnL* spacer is not mentioned as a potential barcode in bryophytes (only as a phylogenetic marker [73] in contrast to the following regions*: trnH-psbA* [20, 70], *atpF-atpH*, *psbK-psbI* [76] and *trnL-trnF* [20, 73]. The *trnH-psbA* spacer is one of a recommended plant DNA barcodes by CBOL Plant Working Group [23]. However, in *Calypogeia* it is not informative like in the *Solidago* genus [77] because of its short length (only 131 bp). As a consequence, *trnH-psbA* is proposed to be used in two- or three-locus barcodes to provide acceptable resolution [77, 78].

Similarly, too short sequences for identification of *Calypogeia* species had the rest of the spacers proposed for bryophytes (71–288 bp). Moreover, it is questionable whether the sequences of the spacers: *atpF-atpH* and *psbK-psbI* could be obtained without problems in a PCR reaction. Low amplification rates of these regions were reported in mosses by Liu et al. [70]. On the other hand, the *trnL-trnF* spacer is reported to be a longer sequence in some liverwort species and amplified with high success [20, 71]. In the genus *Calypogeia*, *trnL-trnF* is only a 71 bp-long region. As a consequence we have tested at least 400 bp-long fragments of non- coding regions according to Hebert et al. [19] who reported that the standard barcode has a length of 400–800 bp. Theoretically, it is possible to apply shorter sequences as DNA barcodes, so- called mini- barcodes (100–250 bp) or even micro- barcodes (within 100 bp) [79, 80]. However, these types of barcodes are rather taxon specific than universal [81]. Currently, it is realistic to search the whole chloroplast genome to find the most informative fragments for species identification. Mini-barcodes for *Calypogeia* should be sought within the *ycf2* gene or between the genes *psbA* and *ycf2* as demonstrated sliding window analysis.

Our research shows that in *Calypogeia* plastome there is a lot of regions which has potential to be barcodes and best match/best close match analysis demonstrated that whole chloroplast genome can be used as a barcode. On the basis of the entire plastome data we revealed that a barcoding gap was present between most of the species. Only one individual was incorrectly identified based on entire plastome sequences as well as based on selected chloroplast regions, namely *C. suecica*. PTP analysis indicated two representatives of *C. suecica* as two separate species (Table 2.), which is in accordance with the results on variability of *Calypogeia* chloroplast genomes. Plastomes of two representatives of *C. suecica* were similar in 97.7%, what indicates quite significant differences taking into account that pairwise identity of all studied plastome sequences of the genus *Calypogeia* was 95.6%. This is probably due to the occurrence of cryptic speciation within *C. suecica*. However, three regions: the *ndhB* and *ndhH* genes and the *trnT*-*trnL* spacer coped very well with solving the riddle about genetic recognition of species (100% of the power discrimination).

The super-barcoding turned out in the case of studied liverwort genus to be slightly less effective (95.45% of the power discrimination) in comparison to a traditional barcoding approach (100% of the power discrimination). However, some of plastid regions with 100% efficiency are very long (*ndhB*- 1, 503 bp; *ndhH*-1, 182 bp) and the amplification of their whole, intact sequences could be problematic. On the other hand, using plastid genome as a marker solves the issues referring to low PCR efficiency or gene loss [82]. Li et al. [27] proposed a new approach to plant DNA barcoding (so-called “1 + 1 Model”) that combines super- and single- locus barcoding. This method consists in a development of the “specific barcode”, which is derived from chloroplast genome of the target plant group and so variable that lets species recognition. Testing 10 the most variable DNA fragments, we found the most specific barcodes for *Calypogeia* species among the protein- coding regions (Table 1). Seven genes correctly assigned 21 out of 22 sequences to the species, two loci (*ycf66*, *rpoC2*)- 20 out of 22 sequences and one locus (*ndhB*) identified rightly all individuals. Protein- coding regions were the least mutable in comparison to non- coding regions and fragments generated by the sliding window approach. The last method allowed to obtain the most variable plastid DNA pieces with length over 400 bp, but unfortunately its efficiency in species discrimination, similar to non- coding regions, was lower. Our results proved that a good barcode may be even a region with average variability like the *ndhH* gene taking the 51. position in the ranking of the most variable protein- coding regions in the genus *Calypogeia*.

## Conclusion

In conclusion, complete plastid sequences applied as a super-barcode for *Calypogeia* are not effective in 100%, nonetheless their success of species discrimination (95.45%) is still conspicuous. The above outcome is probably a result of the cryptic speciation in *C. suecica*. Further studies are required to clear this issue. On the other hand, super- barcoding approach for species identification does not close the door to a traditional single- or multi- locus barcoding. Moreover, it avoids many complication resulting from the need to amplify selected DNA fragments. Having the sequences of entire plastomes of European *Calypogeia* species, we discovered that the *ndhB* and *ndhH* genes and the *trnT-trnL* spacer identify species in 100%. It seems that a good solution for species discrimination is a development of so- called “specific barcodes” for a given taxonomic group, based on plastome data.

## Methods

### Plant material

Plant material used in this study came from the following herbaria: Herbarium of Adam Mickiewicz University, Herbarium D.A. Callaghan and Herbarium Schäfer-Verwimp (Table S1). Twenty-two specimens stood for 10 taxa of *Calypogeia*: *C. integristipula, C. suecica, C. fissa, C. sphagnicola, C. paludosa, C. muelleriana, C. azurea, C. arguta, C. azorica, C. neesiana*. The total genomic DNA from two specimens of each European *Calypogeia* species were extracted using ZR Plant/Seed DNA MiniPrep™ kit (Zymo Research, Irvine, CA, USA). Only two species- *C. paludosa* and *C. muelleriana* were represented by three individuals. DNA quantity was estimated using Qubit fluorometer and Qubit™ dsDNA BR Assay Kit (Invitrogen, Carsbad, NM, USA).

### Plastid genome sequencing, assembly and annotation

The genomic library was constructed with TruSeq Nano DNA kit (Illumina, San Diego, CA, USA) and was sequenced using HiSeqX (Illumina) to generate 150 bp paired-end reads at Macrogen Inc. (Seoul, Korea) with 350 bp insert size between paired-ends. Due to low amount of available plant material of an endemic to Azores *C. azorica* the genomic library had to be constructed using alternative kit which enables lower concentration of input DNA. Sequencing libraries of *C. azorica* were prepared using Qiagen FX library kit according to manufacturer protocol. After sequencing, reads were cleaned by removing the adaptor sequences and low-quality reads with Trimmomatic v0.36 [83]. The filtered reads were de novo assembled using Geneious R8 software [84]. Afterwards, to verify assembly results, the filtered reads were mapped to the reference chloroplast genome of *Ptilidium pulcherrimum* (sequence similarity set to 90%). Next contigs derived after mapping were iteratively mapped (sequence similarity set to 100%) until subsequent iterations did not result with sequence extension. The results of iterative mapping approach were consistent with de novo assembly. The above analyses were performed using Geneious R8 software [84].

Genes were identified and annotated based on the closest known chloroplast genomes: *Aneura pinguis*, *Marchantia paleacea, Pellia endiviifolia, Ptilidium pulcherrimum*. Predictions were made using Geneious R8 software [84] and the BLAST tool [85]. Annotated sequences of *Calypogeia* chloroplast genomes were submitted to GenBank with the accession numbers specified in Table S1. Circular genome map was created using the OGDraw software [86].

### Variation analyzes

Twenty-two chloroplast genomes of 10 *Calypogeia* species were aligned using MAFFT genome aligner [87]. Afterwards based on alignment of plastomes polymorphism analysis was conducted separately for each protein- coding sequence, intron, intergenic spacer and for each 500 bp- long fragment generated by sliding window analysis. Every variation within aforementioned regions was identified as single nucleotide polymorphism (SNP) or insertion/deletion (indel) and counted using custom Phyton script. Each SNP within coding sequence was defined as synonymous or nonsynonymous substitution. Variations (SNPs and indels) were visualized using Circos software [88] combined with Python script. The nucleotide diversity (π) was calculated for each coding and noncoding region using Tassel 5.0 [89] and for each 500 bp-long fragment of plastom the π value was computed in Spider [90]. Because the nucleotide diversity is based only on substitutions, percent of polymorphic sites (P_%_) are given for each region (Table S2, S3, S4).

### Phylogenetic analyzes

Phylogenetic analyses were performed using chloroplast genomes of 27 species: 22 of *Calypogeia* genus and 5 other known liverworts. Out of each plastome sequence CDS of 68 genes, common to all species, were extracted and translated into amino acid sequences. MAFFT software [87] was used to create three alignments: 27 CDS of 68 genes, 27 amino acid sequences of 68 genes and 27 whole plastome sequences. Next, with the use of PartitionFinder2 [91], the best partitioning schemes and corresponding substitution models of each alignment were estimated. Afterwards, based on the alignments and obtained models, Bayesian analysis was conducted using MrBayes 3.2.6 [92]. The MCMC algorithm was run for 5, 000, 000 generations (sampling every 500) with four incrementally heated chains (starting from random trees). The Tracer 1.7.1 [93] software was used to determine the number of generations needed to reach stationarity, which occurred at approximately 300,000 generations. Therefore, the first 600 trees were discarded as burn-in, and the remaining trees were used to develop a Bayesian consensus tree. *Marchantia paleacea* and *Marchantia polymorpha* were used as an outgroup in each of three obtained phylogenetic trees.

### Species delimitation

The Poisson Tree Processes (PTP) method was applied to delimitate species boundaries [94]. The PTP model delimits species using the number of substitutions without the difficult and error prone procedures of time calibration. The fundamental assumption of this analysis is that the number of substitutions between species is significantly higher than the number of substitutions within species. The PTP model places the number of species in a set of query sequences into a specific branch of the reference phylogeny. So it only requires a phylogenetic input tree, for example the output of RAxML - the branch lengths should represent number of mutations. The analysis was performed using a rooted tree, the MCMC algorithm was run for 1,000,000 generations, with 100 thinning and 0.2 burn-in.

Comparative analysis of chloroplast genomes was carried out in DnaSP v6.12 [95] (number of fixed nucleotide differences), using custom PHP script (number of indels differing plastomes of particular species) and in Spider program [90] based on inter- and intraspecific distances that calculated using Kimura 2-parameter model (K2P) of nucleotide substitution [96].

Barcoding analyzes of entire *Calypogeia* plastomes and their 500 bp-long fragments generated by sliding window were made in Spider [90], whereas Best Match/Best Close Match analyzes were performed in Species Identifier 1.8 program from TAXON-DNA software package [97]. The threshold was set to 95% [97]. The latter analysis were carried out for both the whole chloroplast genomes and the most variable protein-coding and non-coding regions of *Calypogeia* plastome with length at least 400 bp in accordance with a definition of plant DNA barcode given by Hebert et al. [19]. We have also tested an usefulness of sequences that were recommended as barcodes by other researchers (Table S5).

## Supplementary information

**Additional file 1: Table S1.** Species used in this study, sampling data and sequencing results.

**Additional file 2: Table S2.** SNP and indel variation within chloroplast genes of *Calypogeia* species.

**Additional file 3: Table S3.** SNP and indel variation within chloroplast noncoding regions of *Calypogeia* species.

**Additional file 4: Table S4.** SNP and indel variation within chloroplast 500-bp fragments of *Calypogeia* species.

**Additional file 5: Table S5.** Discrimination power of the DNA barcodes recommended for plants.

**Additional file 6: Figure S1.** Phylograms based on amino acids and complete plastid genomes of *Calypogeia* species. The posterior probability value lower than 1 is given at the node.

## Data Availability

Supplementary data are available in Supporting Information. All *Calypogeia* plastomes are submitted to GenBank with their accession numbers given in Table S1.

## Abbreviations

CBOL: Consortium for the Barcode of Life
CDS: Coding sequence
cpDNA: chloroplast DNA
IR: Inverted repeat region
LSC: Large single copy region
SSC: Small single copy region
P_%_: Percent of polymorphic sites
