# Supplementary material for: Molecular delimitation of European leafy liverworts of the genus Calypogeia based on plastid super-barcodes
Source: BMC Plant Biol. 2020 May 28;20:243. doi: 10.1186/s12870-020-02435-y (PMC7257191; doi:10.1186/s12870-020-02435-y)
Supplement: Supplementary file 1 — Additional file 1: Table S1. Species used in this study, sampling data and sequencing results. [file 12870_2020_2435_MOESM1_ESM.docx]

**Table S1. Species used in this study, sampling data and sequencing results.**

| **species** | **voucher** | **Geographic coordinates** | **sequencing results** | **plastome**  **length**  **[bp]** | **plastome mean coverage** | **GenBank accession number** |
| --- | --- | --- | --- | --- | --- | --- |
| *Calypogeia arguta* | United Kingdom, coll. D.A.C. DC1420 | No data | 383,522,854 2x 150bp pair-end reads | 119,628 | 24,433.4 | MK293988 |
|  | Spain, Galicia, Province Ourense, Serra Do Xurés National Park, coll. A.S.-V. and I.V.  S-V 31365 | 37.50°N, 25.48°W | 27,129,272 2x 100bp pair-end reads | 119,653 | 400.2 | MK293989 |
| *Calypogeia integristipula* | SE Poland, Bieszczady Mts, W slope of Mt Rozsypaniec Wołosacki, 1214 m, coll. K.B. POZW 41928 | 49.06°N, 22.77°E | 16,913,340 2x 100bp pair-end reads | 120,107 | 198.2 | MK293997 |
|  | S Poland, Tatra Mts, valley of Białka stream, 1100 m a.s.l., coll. K.B., A.B.  POZW 40856 | 49.24°N, 20.10°E | 19,604,234 2x 100bp pair-end reads | 120,137 | 407.7 | MK293996 |
| *Calypogeia fissa* | W Poland, Lubuskie Province, Biecz forestry, coll. S.R., K.B.  POZW 42306 | 51.48°N, 15.10°E | 14,523,216 2x 100bp pair-end reads | 120,087 | 329.5 | MK293995 |
|  | NW Poland, Pomorskie Province, Lake Małe Sitno near Czarna Dąbrówka, coll. K.B., A.B. POZW 42345 | 54.16°N, 17.31°E | 22,987,466 2x 100bp pair-end reads | 120,087 | 275.9 | MK293994 |
| *Calypogeia suecica* | SE Poland, Beskid Sądecki Mts, Potok Czarny stream, 717 m, coll. K.B. POZW 42366 | 49.26°N, 20.28°E | 11,741,230 2x 100bp pair-end reads | 119,946 | 196.7 | MK294009 |
|  | SE Poland, Bieszczady Mts, Górna Solinka Valley, 772 m, coll. K.B. POZW 41937 | 49.12°N, 22.50°E | 12,861,994 2x 100bp pair-end reads | 120,076 | 135.2 | MK294008 |
| *Calypogeia neesiana* | S Poland, Tatra Mts, N slope of Mt Ornak, 1680 m, coll. K.B., A.B. POZW 41731 | 49.13°N, 19.50°E | 16,367,294 2x 100bp pair-end reads | 119,986 | 203.1 | MK294002 |
|  | SE Poland, Bieszczady Mts, W slope of Mt Tarmica, 1280 m, coll. K.B. POZW 41952 | 49.05°N, 22.44°E | 6,008,162 2x 300bp pair-end reads | 119,989 | 78.6 | MK294001 |
| *Calypogeia azurea* | S Poland, Tatra Mts, NE slope of Skupinów Upłaz Mt, 1200 m, coll. K.B., A.B. POZW 41372 | 49.27°N, 19.99°E | 16,375,214 2x 100bp pair-end reads | 120,041 | 477.6 | MK293993 |
|  | S Poland, Tatra Mts, Rów Zakopiański at N base of Tatra Mts, 971 m, coll. A.B., K.B. POZW 41388 | 49.31°N, 20.05°E | 14,787,148 2x 100bp pair-end reads | 120,032 | 303.6 | MK293992 |
| *Calypogeia sphagnicola* | NW Poland, Pomorskie Province, Lake Wałachy near Kościerzyna, coll. K.B., A.B. POZW 42243 | 54.00°N, 17.57°E | 15,389,336 2x 100bp pair-end reads | 119,960 | 74.6 | MK294007 |
|  | S Poland, Row Zakopiański at N base of Tatra Mts, 971 m, coll. K.B., A.B. POZW 41695 | 49.31°N, 20.05°E | 6,784,012 2x 300bp pair-end reads | 119,965 | 89.7 | MK294006 |
| *Calypogeia paludosa* | S Poland, Tatra Mts, lake Toporowy Staw Wyżni, coll. K.B., A.B. POZW 41142 | 49.16°N, 20.01°E | 13,411,696 2x 100bp pair-end reads | 120,053 | 429.7 | MK294004 |
|  | S Poland, Tatra Mts, E slope of Mt. Żołta Turnia, 1687 m, coll. K.B., A.B. POZW 41178 | 49.14°N, 20.00°E | 13,801,740 2x 100bp pair-end reads | 120,052 | 159.3 | MK294005 |
|  | S Poland, Tatra Mts, Pańszczyca Valley, peat bog Wielka Pańszczycka Młaka, 1,274 m a.s.l. K.B., A.B. POZW 41173 | No data | 12,689,684 2x 100bp pair-end reads | 120,033 | 118.6 | MK294003 |
| *Calypogeia muelleriana* | W Poland, Lubuskie Province, Biecz forestry, coll. S.R., K.B. POZW 42318 | 51.48°N, 15.10°E | 388,599,830 2x 150bp pair-end reads | 120,170 | 18,346.3 | MK293998 |
|  | NW Poland, Pomorskie Province, Lake Orle near Miastko, coll. K.B., A.B. POZW 41346 | 54.01°N, 17.04°E | 11,588.688 2x 100bp pair-end reads | 120,128 | 85.1 | MK294000 |
|  | NW Poland, Pomorskie Province, Lake Lubygość near Kartuzy, coll. K.B., A.B. POZW 42220 | 54.24°N, 17.59°E | 12,280,120 2x 100bp pair-end reads | 120,137 | 237.1 | MK293999 |
| *Calypogeia azorica* | Portugal, Azores, Sao Migiel Island, coll. A.S.-V. and I.V. S-V 29154 | 37.50°N, 25.48°W | 31,811,750 2x 150bp pair-end reads | 119,920 | 347.8 | MK293990 |
|  | Portugal, Azores, Sao Migiel Island, coll. A.S.-V. and I.V. S-V 29425 | 37.44°N, 25.18°W | 30,016,558 2x 150bp pair-end reads | 119,920 | 188.3 | MK293991 |

**Collectors:** A.B. – Alina Bączkiewicz, A.S. – A. Sloga, A.S.-V. – Alfons Schäfer-Verwimp, I.V. – I. Verwimp, K.B. – Katarzyna Buczkowska, D.A.C. – Des A. Callaghan, D.Q. – D. Quandt, S.R. – Stanisław Rosadziński; **Herbaria:** DC – Herb. D. A. Callaghan, POZW – Herbarium of Adam Mickiewicz University, S -V – Herb. Schäfer-Verwimp
