# Supplementary material for: Molecular delimitation of European leafy liverworts of the genus Calypogeia based on plastid super-barcodes
Source: BMC Plant Biol. 2020 May 28;20:243. doi: 10.1186/s12870-020-02435-y (PMC7257191; doi:10.1186/s12870-020-02435-y)
Supplement: Supplementary file 2 — Additional file 2: Table S2. SNP and indel variation within chloroplast genes of Calypogeia species. [file 12870_2020_2435_MOESM2_ESM.docx]

**Table S2. SNP and indel variation within chloroplast genes of *Calypogeia*species*.***

Table represents SNP and indel occurrence within chloroplast genes including nonsynonymous SNPs. P_%_ - percent of polymorphic sites (percent of SNPs and indels per CDS length), π - nucleotide diversity. Genes sorted by P_%_.

| **Gene** | **Start** | **End** | **Length**  **[bp]** | **SNP** | **Indel** | **Nonsynonymous** | **P_%_**  **[%]** | **π** |
| --- | --- | --- | --- | --- | --- | --- | --- | --- |
| ***cysT*** | 96414 | 97282 | 867 | 164 | 15 | 92 | 20.64590542 | 0.07389 |
| ***ycf2*** | 31502 | 37741 | 6216 | 1229 | 25 | 782 | 20.17374517 | 0.06018 |
| ***matK*** | 27957 | 29057 | 1101 | 218 | 1 | 121 | 19.89100817 | 0.05696 |
| ***ycf66*** | 4480 | 5487 | 531 | 100 | 4 | 68 | 19.58568738 | 0.07282 |
| ***ycf1*** | 107755 | 110913 | 3147 | 549 | 3 | 299 | 17.54051478 | 0.04912 |
| ***rpl32*** | 96054 | 96254 | 201 | 35 | 0 | 14 | 17.41293532 | 0.04483 |
| ***psbK*** | 24230 | 24403 | 174 | 30 | 0 | 8 | 17.24137931 | 0.04198 |
| ***rpoC2*** | 12290 | 16462 | 4167 | 704 | 2 | 349 | 16.94264459 | 0.04721 |
| ***ndhB*** | 1667 | 3815 | 1503 | 248 | 1 | 96 | 16.56686627 | 0.04557 |
| ***ndhF*** | 93100 | 95214 | 2112 | 340 | 2 | 154 | 16.19318182 | 0.04739 |
| ***cysA*** | 38489 | 39596 | 1092 | 164 | 12 | 90 | 16.11721612 | 0.056 |
| ***rpl23*** | 83707 | 83982 | 276 | 44 | 0 | 19 | 15.94202899 | 0.04836 |
| ***rpl36*** | 78252 | 78365 | 114 | 18 | 0 | 2 | 15.78947368 | 0.04755 |
| ***rpl20*** | 68517 | 68873 | 357 | 56 | 0 | 23 | 15.68627451 | 0.03991 |
| ***ndhG*** | 101429 | 102016 | 588 | 90 | 1 | 39 | 15.47619048 | 0.04518 |
| ***psaC*** | 100648 | 100893 | 246 | 38 | 0 | 20 | 15.44715447 | 0.04593 |
| ***rpl22*** | 81586 | 81948 | 363 | 55 | 0 | 22 | 15.15151515 | 0.04097 |
| ***rps15*** | 105776 | 106053 | 267 | 39 | 1 | 16 | 14.98127341 | 0.04062 |
| ***rpoB*** | 6222 | 9417 | 3195 | 469 | 1 | 136 | 14.71048513 | 0.0391 |
| ***cemA*** | 62726 | 64075 | 1347 | 196 | 2 | 86 | 14.69933185 | 0.03844 |
| ***rpl2*** | 82279 | 83672 | 831 | 119 | 1 | 41 | 14.44043321 | 0.03873 |
| ***ycf4*** | 62070 | 62624 | 555 | 79 | 0 | 24 | 14.23423423 | 0.0358 |
| ***ndhK*** | 53866 | 54618 | 753 | 107 | 0 | 50 | 14.20982736 | 0.04204 |
| ***ndhC*** | 54609 | 54971 | 363 | 51 | 0 | 18 | 14.04958678 | 0.03548 |
| ***rps4*** | 51497 | 52105 | 609 | 83 | 0 | 20 | 13.62889984 | 0.03351 |
| ***ndhJ*** | 53331 | 53843 | 507 | 68 | 1 | 20 | 13.60946746 | 0.03748 |
| ***rpoC1*** | 9455 | 12185 | 2082 | 282 | 1 | 93 | 13.59269933 | 0.0362 |
| ***rps3*** | 80890 | 81546 | 657 | 89 | 0 | 23 | 13.54642314 | 0.03821 |
| ***ndhE*** | 101051 | 101353 | 303 | 41 | 0 | 6 | 13.53135314 | 0.03337 |
| ***rps7*** | 1062 | 1529 | 468 | 63 | 0 | 13 | 13.46153846 | 0.04058 |
| ***rpl21*** | 95566 | 95922 | 357 | 48 | 0 | 17 | 13.44537815 | 0.03177 |
| ***ccsA*** | 97878 | 98837 | 960 | 128 | 0 | 57 | 13.33333333 | 0.03378 |
| ***rpoA*** | 76762 | 77772 | 1011 | 134 | 0 | 47 | 13.25420376 | 0.03457 |
| ***rpl16*** | 79783 | 80834 | 426 | 56 | 0 | 10 | 13.14553991 | 0.03137 |
| ***chlL*** | 112491 | 113363 | 873 | 114 | 0 | 20 | 13.05841924 | 0.03505 |
| ***rps11*** | 77805 | 78197 | 393 | 51 | 0 | 6 | 12.97709924 | 0.03116 |
| ***psbN*** | 73655 | 73786 | 132 | 17 | 0 | 2 | 12.87878788 | 0.03317 |
| ***rps12*** | 249 | 1009 | 258 | 33 | 0 | 5 | 12.79069767 | 0.03729 |
| ***rps2*** | 16650 | 17364 | 714 | 89 | 1 | 17 | 12.60504202 | 0.03381 |
| ***ndhI*** | 102106 | 102654 | 549 | 67 | 1 | 23 | 12.38615665 | 0.03518 |
| ***ndhD*** | 99035 | 100543 | 1509 | 186 | 0 | 62 | 12.32604374 | 0.0307 |
| ***rps19*** | 81966 | 82244 | 279 | 34 | 0 | 6 | 12.18637993 | 0.02884 |
| ***chlN*** | 111034 | 112434 | 1401 | 170 | 0 | 38 | 12.13418986 | 0.03022 |
| ***rps18*** | 68205 | 68432 | 228 | 27 | 0 | 8 | 11.84210526 | 0.02869 |
| ***chlB*** | 24899 | 26440 | 1542 | 182 | 0 | 34 | 11.80285344 | 0.03011 |
| ***rps8*** | 78725 | 79123 | 399 | 47 | 0 | 11 | 11.77944862 | 0.03122 |
| ***atpE*** | 56245 | 56661 | 417 | 49 | 0 | 13 | 11.75059952 | 0.03034 |
| ***petA*** | 64283 | 65245 | 963 | 112 | 0 | 32 | 11.63032191 | 0.02922 |
| ***petB*** | 74231 | 75472 | 648 | 75 | 0 | 6 | 11.57407407 | 0.0279 |
| ***ndhA*** | 102769 | 104574 | 1110 | 124 | 0 | 45 | 11.17117117 | 0.0294 |
| ***ndhH*** | 104576 | 105757 | 1182 | 132 | 0 | 18 | 11.16751269 | 0.02916 |
| ***petN*** | 5482 | 5571 | 90 | 10 | 0 | 4 | 11.11111111 | 0.02825 |
| ***psbT*** | 73478 | 73585 | 108 | 12 | 0 | 1 | 11.11111111 | 0.02096 |
| ***psbH*** | 73883 | 74107 | 225 | 25 | 0 | 6 | 11.11111111 | 0.02982 |
| ***rbcL*** | 58761 | 60188 | 1428 | 157 | 0 | 25 | 10.99439776 | 0.02836 |
| ***rps14*** | 44012 | 44314 | 303 | 33 | 0 | 9 | 10.89108911 | 0.02991 |
| ***rpl14*** | 79292 | 79661 | 369 | 39 | 1 | 4 | 10.8401084 | 0.02519 |
| ***psaI*** | 61743 | 61853 | 111 | 12 | 0 | 4 | 10.81081081 | 0.02532 |
| ***psaB*** | 44365 | 46569 | 2205 | 238 | 0 | 38 | 10.79365079 | 0.0283 |
| ***petD*** | 75616 | 76622 | 483 | 52 | 0 | 10 | 10.76604555 | 0.02749 |
| ***accD*** | 60620 | 61576 | 957 | 102 | 1 | 22 | 10.76280042 | 0.02875 |
| ***clpP*** | 69746 | 71387 | 615 | 66 | 0 | 12 | 10.73170732 | 0.02649 |
| ***atpF*** | 19213 | 20337 | 555 | 59 | 0 | 14 | 10.63063063 | 0.02787 |
| ***psbJ*** | 65386 | 65508 | 123 | 13 | 0 | 2 | 10.56910569 | 0.03042 |
| ***infA*** | 78402 | 78638 | 237 | 25 | 0 | 4 | 10.54852321 | 0.02962 |
| ***atpB*** | 56669 | 58147 | 1479 | 155 | 0 | 20 | 10.48005409 | 0.02596 |
| ***psbB*** | 71800 | 73317 | 1518 | 159 | 0 | 17 | 10.4743083 | 0.02698 |
| ***atpA*** | 20381 | 21904 | 1524 | 156 | 1 | 15 | 10.30183727 | 0.02495 |
| ***atpI*** | 17570 | 18310 | 741 | 76 | 0 | 21 | 10.25641026 | 0.02628 |
| ***rpl33*** | 67977 | 68174 | 198 | 20 | 0 | 5 | 10.1010101 | 0.02685 |
| ***psbC*** | 41478 | 42899 | 1422 | 141 | 0 | 13 | 9.915611814 | 0.02483 |
| ***petG*** | 67034 | 67147 | 114 | 11 | 0 | 2 | 9.649122807 | 0.02295 |
| ***psbM*** | 4236 | 4340 | 105 | 10 | 0 | 2 | 9.523809524 | 0.02165 |
| ***psbL*** | 65629 | 65745 | 117 | 11 | 0 | 1 | 9.401709402 | 0.02245 |
| ***psbI*** | 23831 | 23941 | 111 | 10 | 0 | 3 | 9.009009009 | 0.02423 |
| ***psbD*** | 40469 | 41530 | 1062 | 94 | 0 | 9 | 8.851224105 | 0.02173 |
| ***ycf12*** | 22947 | 23051 | 105 | 8 | 1 | 2 | 8.571428571 | 0.03088 |
| ***atpH*** | 18697 | 18942 | 246 | 18 | 0 | 9 | 7.317073171 | 0.023 |
| ***psaA*** | 46596 | 48848 | 2253 | 162 | 0 | 15 | 7.190412783 | 0.01763 |
| ***psaJ*** | 67683 | 67811 | 129 | 9 | 0 | 2 | 6.976744186 | 0.01885 |
| ***psbZ*** | 43223 | 43411 | 189 | 12 | 0 | 3 | 6.349206349 | 0.01618 |
| ***petL*** | 66816 | 66911 | 96 | 6 | 0 | 2 | 6.25 | 0.01195 |
| ***ycf3*** | 49187 | 51063 | 504 | 31 | 0 | 6 | 6.150793651 | 0.01586 |
| ***psbA*** | 29324 | 30385 | 1062 | 55 | 0 | 10 | 5.178907721 | 0.01441 |
| ***psbE*** | 65901 | 66152 | 252 | 12 | 0 | 0 | 4.761904762 | 0.01057 |
| ***psaM*** | 23293 | 23391 | 99 | 4 | 0 | 0 | 4.04040404 | 0.00914 |
| ***psbF*** | 65769 | 65888 | 120 | 3 | 0 | 0 | 2.5 | 0.00474 |
