# Supplementary material for: Molecular delimitation of European leafy liverworts of the genus Calypogeia based on plastid super-barcodes
Source: BMC Plant Biol. 2020 May 28;20:243. doi: 10.1186/s12870-020-02435-y (PMC7257191; doi:10.1186/s12870-020-02435-y)
Supplement: Supplementary file 3 — Additional file 3: Table S3. SNP and indel variation within chloroplast noncoding regions of Calypogeia species. [file 12870_2020_2435_MOESM3_ESM.docx]

**Table S3. SNP and indel variation within chloroplast noncoding regions of *Calypogeia*species.**

Table represents SNP and indel occurrence within chloroplast non-coding regions. P_%_ - percent of polymorphic sites (percent of SNPs and indels per region length), π - nucleotide diversity. Noncoding regions sorted by P_%_.

| **Noncoding region** | **Start** | **End** | **Length**  **[bp]** | **SNP** | **Indel** | **P_%_**  **[%]** | **π** |
| --- | --- | --- | --- | --- | --- | --- | --- |
| ***ndhE-ndhG*** | 101354 | 101428 | 75 | 25 | 2 | 36 | 0.15474 |
| ***psbF-psbE*** | 65889 | 65900 | 12 | 3 | 1 | 33.3333333 | 0.06316 |
| ***ndhH-rps15*** | 105758 | 105775 | 18 | 5 | 1 | 33.3333333 | 0.05414 |
| ***rpl14-rpl16*** | 79662 | 79782 | 121 | 30 | 6 | 29.7520661 | 0.12371 |
| ***rps11-rpl36*** | 78198 | 78251 | 54 | 13 | 3 | 29.6296296 | 0.09055 |
| ***rpl32-cysT*** | 96255 | 96413 | 159 | 43 | 3 | 28.9308176 | 0.09156 |
| ***atpH-atpF*** | 18943 | 19212 | 270 | 67 | 11 | 28.8888889 | 0.11377 |
| ***petD-rpoA*** | 76623 | 76761 | 139 | 33 | 7 | 28.7769784 | 0.14211 |
| ***atpE-atpB*** | 56662 | 56668 | 7 | 2 | 0 | 28.5714286 | 0.09925 |
| ***rpl36-infA*** | 78366 | 78401 | 36 | 9 | 1 | 27.7777778 | 0.07544 |
| ***ccsA-ndhD*** | 98838 | 99034 | 197 | 51 | 3 | 27.4111675 | 0.09818 |
| ***ycf4-cemA*** | 62625 | 62725 | 101 | 21 | 6 | 26.7326733 | 0.08551 |
| ***rpl2-rpl23*** | 83673 | 83706 | 34 | 6 | 3 | 26.4705882 | 0.07028 |
| ***petB-petD*** | 75473 | 75615 | 143 | 28 | 9 | 25.8741259 | 0.08811 |
| ***ndhI-ndhA*** | 102655 | 102768 | 114 | 24 | 5 | 25.4385965 | 0.11011 |
| ***cemA-petA*** | 64076 | 64282 | 207 | 47 | 5 | 25.1207729 | 0.10005 |
| ***rps8-rpl14*** | 79124 | 79291 | 168 | 37 | 5 | 25 | 0.25188 |
| ***psbH-petB*** | 74108 | 74230 | 123 | 30 | 0 | 24.3902439 | 0.06397 |
| ***ycf66 intron1*** | 4585 | 5053 | 469 | 106 | 8 | 24.3070362 | 0.07038 |
| ***rpoC2-rps2*** | 16463 | 16649 | 187 | 40 | 5 | 24.0641711 | 0.08463 |
| ***ndhG-ndhI*** | 102017 | 102105 | 89 | 20 | 1 | 23.5955056 | 0.06168 |
| ***rbcL-accD*** | 60189 | 60619 | 431 | 87 | 12 | 22.9698376 | 0.09565 |
| ***rpl21-rpl32*** | 95923 | 96053 | 131 | 25 | 5 | 22.9007634 | 0.10346 |
| ***rps7-ndhB*** | 1530 | 1666 | 137 | 25 | 6 | 22.6277372 | 0.07733 |
| ***psbI-psbK*** | 23942 | 24229 | 288 | 61 | 4 | 22.5694444 | 0.10779 |
| ***psbJ-psbL*** | 65509 | 65628 | 120 | 25 | 2 | 22.5 | 0.06342 |
| ***petB intron1*** | 74237 | 74830 | 594 | 119 | 14 | 22.3905724 | 0.0775 |
| ***psbA-ycf2*** | 30386 | 31501 | 1116 | 227 | 22 | 22.311828 | 0.1507 |
| ***petA-psbJ*** | 65246 | 65385 | 140 | 25 | 6 | 22.1428571 | 0.10143 |
| ***rpoB-rpoC1*** | 9418 | 9454 | 37 | 7 | 1 | 21.6216216 | 0.06013 |
| ***psaM-psbI*** | 23392 | 23830 | 439 | 83 | 11 | 21.4123007 | 0.07987 |
| ***psaI-ycf4*** | 61854 | 62069 | 216 | 41 | 5 | 21.2962963 | 0.07973 |
| ***psaJ-rpl33*** | 67812 | 67976 | 165 | 30 | 4 | 20.6060606 | 0.10354 |
| ***ndhB intron1*** | 2393 | 3035 | 643 | 117 | 13 | 20.2177294 | 0.06377 |
| ***ndhC-atpE*** | 54972 | 56244 | 1273 | 235 | 22 | 20.188531 | 0.09266 |
| ***petN-rpoB*** | 5572 | 6221 | 650 | 123 | 8 | 20.1538462 | 0.05905 |
| ***rps12 intron1*** | 481 | 983 | 503 | 91 | 10 | 20.0795229 | 0.06037 |
| ***rpoC1 intron1*** | 9887 | 10535 | 649 | 117 | 13 | 20.0308166 | 0.0653 |
| ***psbE-petL*** | 66153 | 66815 | 663 | 124 | 8 | 19.9095023 | 0.06448 |
| ***psbK-chlB*** | 24404 | 24898 | 495 | 89 | 6 | 19.1919192 | 0.09607 |
| ***clpP intron2*** | 69998 | 70487 | 490 | 84 | 9 | 18.9795918 | 0.07011 |
| ***chlB-matK*** | 26441 | 27956 | 1516 | 267 | 19 | 18.8654354 | 0.06449 |
| ***psbB-psbT*** | 73318 | 73477 | 160 | 26 | 4 | 18.75 | 0.04464 |
| ***psbM-ycf66*** | 4341 | 4479 | 139 | 24 | 2 | 18.705036 | 0.0749 |
| ***rps15-ycf1*** | 106054 | 107754 | 1701 | 305 | 12 | 18.6360964 | 0.04957 |
| ***cysA-psbD*** | 39597 | 40468 | 872 | 149 | 13 | 18.5779817 | 0.06934 |
| ***ndhF-rpl21*** | 95215 | 95565 | 351 | 60 | 5 | 18.5185185 | 0.05118 |
| ***petG-psaJ*** | 67148 | 67682 | 535 | 86 | 11 | 18.1308411 | 0.06104 |
| ***rps4-ndhJ*** | 52106 | 53330 | 1225 | 200 | 22 | 18.122449 | 0.07469 |
| ***ycf2-cysA*** | 37742 | 38488 | 747 | 121 | 14 | 18.0722892 | 0.05921 |
| ***atpB-rbcL*** | 58148 | 58760 | 613 | 99 | 11 | 17.9445351 | 0.05661 |
| ***rps18-rpl20*** | 68433 | 68516 | 84 | 15 | 0 | 17.8571429 | 0.0515 |
| ***cysT-ccsA*** | 97283 | 97877 | 595 | 94 | 12 | 17.8151261 | 0.08914 |
| ***chlL-rps12*** | 113364 | 248 | 452 | 76 | 4 | 17.699115 | 0.06177 |
| ***rpl16 intron1*** | 80200 | 80825 | 626 | 98 | 12 | 17.571885 | 0.0545 |
| ***accD-psaI*** | 61577 | 61742 | 166 | 26 | 3 | 17.4698795 | 0.06579 |
| ***rps12-rps7*** | 1010 | 1061 | 52 | 9 | 0 | 17.3076923 | 0.03391 |
| ***petD intron1*** | 75622 | 76145 | 524 | 79 | 11 | 17.1755725 | 0.04798 |
| ***ycf3-rps4*** | 51064 | 51496 | 433 | 65 | 9 | 17.0900693 | 0.07404 |
| ***psbZ-rps14*** | 43412 | 44011 | 600 | 81 | 21 | 17 | 0.10026 |
| ***rpl2 intron1*** | 82713 | 83275 | 563 | 83 | 11 | 16.69627 | 0.05714 |
| ***clpP-psbB*** | 71388 | 71799 | 412 | 63 | 5 | 16.5048544 | 0.0777 |
| ***rpl16-rps3*** | 80835 | 80889 | 55 | 6 | 3 | 16.3636364 | 0.05971 |
| ***rpoC1-rpoC2*** | 12186 | 12289 | 104 | 15 | 2 | 16.3461538 | 0.03897 |
| ***atpI-atpH*** | 18311 | 18696 | 386 | 56 | 7 | 16.3212435 | 0.05899 |
| ***atpA-ycf12*** | 21905 | 22946 | 1042 | 152 | 17 | 16.21881 | 0.05246 |
| ***psbT-psbN*** | 73586 | 73654 | 69 | 8 | 3 | 15.942029 | 0.05706 |
| ***psaC-ndhE*** | 100894 | 101050 | 157 | 23 | 2 | 15.9235669 | 0.04318 |
| ***rpl20-clpP*** | 68874 | 69745 | 872 | 126 | 12 | 15.8256881 | 0.05041 |
| ***petL-petG*** | 66912 | 67033 | 122 | 16 | 3 | 15.5737705 | 0.04206 |
| ***rps2-atpI*** | 17365 | 17569 | 205 | 27 | 4 | 15.1219512 | 0.04475 |
| ***ycf3 intron2*** | 49337 | 49993 | 657 | 92 | 7 | 15.0684932 | 0.05387 |
| ***ycf3 intron1*** | 50222 | 50937 | 716 | 97 | 10 | 14.9441341 | 0.04433 |
| ***psbC-psbZ*** | 42900 | 43222 | 323 | 44 | 4 | 14.8606811 | 0.04585 |
| ***ndhD-psaC*** | 100544 | 100647 | 104 | 13 | 2 | 14.4230769 | 0.03937 |
| ***chlN-chlL*** | 112435 | 112490 | 56 | 8 | 0 | 14.2857143 | 0.039 |
| ***ycf1-chlN*** | 110914 | 111033 | 120 | 14 | 3 | 14.1666667 | 0.04487 |
| ***clpP intron1*** | 70780 | 71316 | 537 | 71 | 5 | 14.1527002 | 0.04015 |
| ***atpF intron1*** | 19354 | 19923 | 570 | 72 | 8 | 14.0350877 | 0.04092 |
| ***ndhJ-ndhK*** | 53844 | 53865 | 22 | 3 | 0 | 13.6363636 | 0.05239 |
| ***psaA-ycf3*** | 48849 | 49186 | 338 | 43 | 3 | 13.6094675 | 0.05186 |
| ***ndhA intron1*** | 103323 | 104018 | 696 | 86 | 7 | 13.362069 | 0.03783 |
| ***ycf12-psaM*** | 23052 | 23292 | 241 | 27 | 5 | 13.2780083 | 0.03923 |
| ***ndhB-psbM*** | 3816 | 4235 | 420 | 52 | 3 | 13.0952381 | 0.04142 |
| ***infA-rps8*** | 78639 | 78724 | 86 | 11 | 0 | 12.7906977 | 0.03807 |
| ***rpoA-rps11*** | 77773 | 77804 | 32 | 2 | 2 | 12.5 | 0.04145 |
| ***rps14-psaB*** | 44315 | 44364 | 50 | 5 | 1 | 12 | 0.03021 |
| ***rps19-rpl2*** | 82245 | 82278 | 34 | 3 | 1 | 11.7647059 | 0.02523 |
| ***matK-psbA*** | 29058 | 29323 | 266 | 30 | 0 | 11.2781955 | 0.02545 |
| ***rpl33-rps18*** | 68175 | 68204 | 30 | 3 | 0 | 10 | 0.01596 |
| ***rps3-rpl22*** | 81547 | 81585 | 39 | 2 | 1 | 7.69230769 | 0.02267 |
| ***psbN-psbH*** | 73787 | 73882 | 96 | 7 | 0 | 7.29166667 | 0.01595 |
| ***rpl23-ndhF*** | 83983 | 93099 | 9117 | 499 | 76 | 6.3068992 | 0.03005 |
| ***rpl22-rps19*** | 81949 | 81965 | 17 | 1 | 0 | 5.88235294 | 0.01115 |
| ***psbL-psbF*** | 65746 | 65768 | 23 | 1 | 0 | 4.34782609 | 0.01648 |
| ***atpF-atpA*** | 20338 | 20380 | 43 | 1 | 0 | 2.3255814 | 0.00441 |
| ***psaB-psaA*** | 46570 | 46595 | 26 | 0 | 0 | 0 | 0 |
| ***ndhA-ndhH*** | 104575 | 104575 | 1 | 0 | 0 | 0 | 0 |
