# Supplementary material for: Molecular delimitation of European leafy liverworts of the genus Calypogeia based on plastid super-barcodes
Source: BMC Plant Biol. 2020 May 28;20:243. doi: 10.1186/s12870-020-02435-y (PMC7257191; doi:10.1186/s12870-020-02435-y)
Supplement: Supplementary file 5 — Additional file 5: Table S5. Discrimination power of the DNA barcodes recommended for plants. [file 12870_2020_2435_MOESM5_ESM.docx]

**Table S5. The discrimination power of the DNA barcodes recommended for plants.**

| **Lp.** | **Barcode** | **References*** | ***Discrimination power*** †  ***[%]*** |
| --- | --- | --- | --- |
| 1. | *matK* | [23] | 95.45% |
| 2. | *rbcL* | [23, 70, 81] | 90.9% |
| 3. | *rpoA* | [22] | 90.9% |
| 4. | *rpoB* | [22, 23, 70, 78] | 95.45% |
| 5. | *rpoC1* | [23, 70, 78] | 95.45% |
| 6. | *rpoC2* | [22, 67, 68, 69] | 90.9% |
| 7. | *ndhF* | [28, 99] | 95.45% |
| **8.** | ***ndhH*** | [28] | **100 %** |
| 9. | *rpl16* | [98] | 95.45% |
| 10. | *rps3* | [99] | 90.9% |
| 11. | *rps4* | [70] | 95.45% |
| 12. | *trnK* | [28] | 90.9% |
| 13. | *ycf1* | [61, 64] | 95.45% |
| 14. | *ycf2* | [29] | 95.45% |
| 15. | *rpl23* | [28] | No match-only 276 bp |
| 16. | *psbA* | [71, 72] | 81.81% |
| 17. | *ndhF-rpl32 (ndhF+rpl21-rpl32 in Calypogeia)* | [28] | 95.45% |
| 18. | *rpl32-ccsA (rpl32-cysT-ccsA u Calypogeia)* | [28] | 81.81% |
| 19. | *trnH-psbA* | [20, 23, 70, 78] | No match - only 131 bp |
| 20. | *trnL-trnF* | [20, 70, 73] | No match- only 71 bp |
| **21.** | ***trnT-trnL*** | [28, 73] | **100%** |
| 22. | *rpl32-trnL*  *(rpl32-cysT-trnL in Calypogeia)* | [75, 99] | 81.81% |
| 23. | *atpF-atpH* | [23, 76] | No match – only 270 bp |
| 24. | *psbK-psbI* | [23, 28, 76] | No match – only 288 bp |
| 25. | *matK+ trnH-psbA* | [22] | 90.9% |
| 26. | *matK+ rpoA* |  |  |
| 27. | *matK + rbcL* | [23] | 95.45% |
| 28. | *trnL-trnF+trnH-psbA* | [20] | No match – only 202 bp |
| 29. | *petA-psbJ* | [28] | No match – only 140 bp |

† percent of correctly identified sequences

*Numbers of references according to the main manuscript reference list.

98. Cialdella AM, Giussani LM, Aagesen L, Zuloaga FO, Morrone O. A phylogeny of *Piptochaetium* (Poaceae: Pooideae: Stipeae) and related genera based on a combined analysis including *trnL-F*, *rpl16*, and morphology. Sys Bot. 2007; 32(3):545-559; doi: 10.1600/036364407782250607.

99. Romaschenko K, Peterson PM, Robert J, Soreng RJ, Garcia-Jacas N, Futorna O, Susanna A. Systematics and evolution of the needle grasses (Poaceae: Pooideae: Stipeae) based on analysis of multiple chloroplast loci, ITS, and lemma micromorphology. Taxon. 2012; 61(1):18-44; doi: 10.1002/tax.611002.
