# Supplementary material for: Molecular delimitation of European leafy liverworts of the genus Calypogeia based on plastid super-barcodes
Source: BMC Plant Biol. 2020 May 28;20:243. doi: 10.1186/s12870-020-02435-y (PMC7257191; doi:10.1186/s12870-020-02435-y)

Figure S1. Phylograms based on amino acids and complete plastid genomes of *Calypogeia* species. The posterior probability value lower than 1 is given at the node.

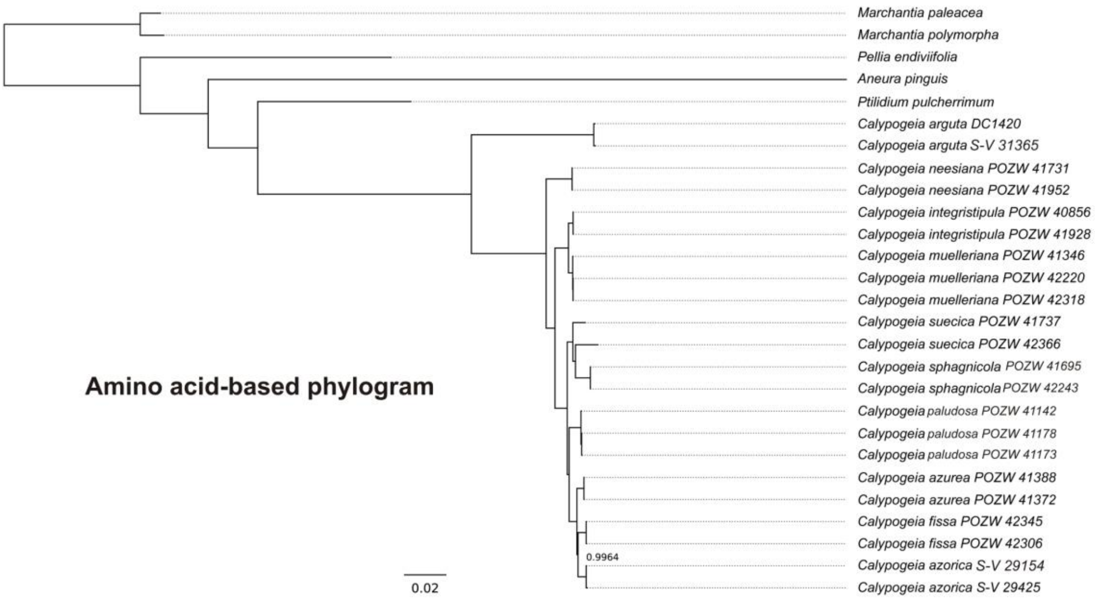

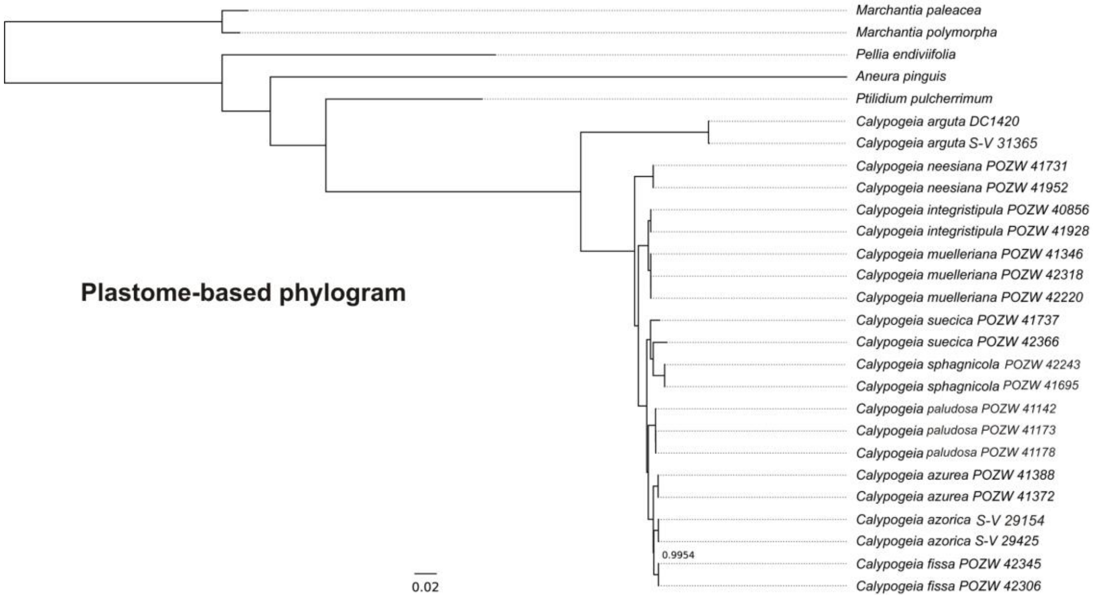

Supplement: Supplementary file 6 — Additional file 6: Figure S1. Phylograms based on amino acids and complete plastid genomes of Calypogeia species. The posterior probability value lower than 1 is given at the node. [file 12870_2020_2435_MOESM6_ESM.pdf]
